# Supplementary material for: The impact of changes in diagnostic testing practices on estimates of COVID-19 transmission in the United States
Source: medRxiv. 2020 Apr 24:2020.04.20.20073338. Preprint. [Version 1] doi: 10.1101/2020.04.20.20073338 (PMC7276051; doi:10.1101/2020.04.20.20073338)
Supplement: 1 [file NIHPP2020.04.20.20073338-supplement-1.pdf]

medRxiv preprint doi: https://doi.org/10.1101/2020.04.20.20073338.this version posted April 24, 2020. The copyright holder for this preprint (which was not certified by peer review) is the author/funder, who has granted medRxiv a license to display the preprint in perpetuity.
It is made available under a CC-BY 4.0 International license .

Supporting Information

Table S1. Scenarios for simulated changes in testing practices.

Scenario	Fraction of true cases detected and reported (p_{test})	Testing capacity, i.e. number of individuals tested for each true case (n_{test})	Reporting delay distribution parameters
1. Constant testing effort	0.5	5	*a*=1.85, *b*=3.57
2. Linear increase in testing probability	0.05 on *d*=20 to 0.15 on *d*=60 in increments of 0.01	5	*a*=1.85, *b*=3.57
3. Linear decrease in testing probability	0.15 on *d*=20 to 0.05 on *d*=60 in increments of 0.01	5	*a*=1.85, *b*=3.57
4. Linear increase in testing capacity	0.5	0.2 on *d*=20 to 0.8 on *d*=60 in increments of 0.015	*a*=1.85, *b*=3.57
5. Linear decrease in testing capacity	0.5	0.8 on *d*=20 to 0.2 on *d*=60 in increments of 0.015	*a*=1.85, *b*=3.57
6. Abrupt increase in testing probability	0.05 for $d \leq 30$<br>0.15 for $d > 30$	5	*a*=1.85, *b*=3.57
7. Abrupt decrease in testing probability	0.15 for $d \leq 30$<br>0.05 for $d > 30$	5	*a*=1.85, *b*=3.57
8. Abrupt increase in testing capacity	0.5	0.2 for $d \leq 30$<br>0.8 for $d > 30$	*a*=1.85, *b*=3.57
9. Abrupt decrease in testing capacity	0.5	0.8 for $d \leq 30$<br>0.2 for $d > 30$	*a*=1.85, *b*=3.57
10. Increase in reporting delay	0.5	5	*a*=1.85 for $d \leq 30$<br>*a*=3.7 for $d > 30$<br>*b*=3.57
11. Decrease in reporting delay	0.5	5	*a*=1.85 for $d \leq 30$<br>*a*=0.925 for $d > 30$<br>*b*=3.57

medRxiv preprint doi: https://doi.org/10.1101/2020.04.20.20073338.this version posted April 24, 2020. The copyright holder for this preprint (which was not certified by peer review) is the author/funder, who has granted medRxiv a license to display the preprint in perpetuity.
It is made available under a CC-BY 4.0 International license .

Table S2. Estimates of the basic reproductive number, R_0, for different US states. Estimates are based on fitting the growth rate in the number of confirmed cases or total number of tests performed through March 24, 2020, using Poisson regression, and assume a fixed serial interval of 6.5 days. The 95% confidence intervals (CI) incorporate only uncertainty in the estimated growth rate.

State abbrev.	R_0 (based on confirmed cases)	95% CI (cases) lower bound	95% CI (cases) upper bound	R_0 (based on number of tests)	95% CI (tests) lower bound	95% CI (tests) upper bound
**AK**	4.25	3.47	5.23	3.05	2.98	3.12
**AL**	3.58	3.33	3.87	7.17	6.95	7.40
**AR**	3.72	3.46	4.01	3.59	3.50	3.69
**AZ**	4.16	3.91	4.40	2.33	2.26	2.39
**CA**	2.69	2.65	2.74	2.58	2.57	2.60
**CO**	3.01	2.92	3.10	3.12	3.08	3.15
**CT**	4.72	4.49	4.95	5.04	4.96	5.12
**DC**	3.23	2.99	3.49	4.02	3.91	4.12
**DE**	3.37	3.01	3.75	2.04	1.92	2.16
**FL**	3.60	3.51	3.69	4.19	4.15	4.22
**GA**	3.53	3.43	3.63	2.89	2.82	2.95
**HI**	3.71	3.30	4.18	11.86	11.46	12.30
**IA**	2.72	2.50	2.93	4.41	4.31	4.50
**ID**	3.63	3.06	4.25	3.19	3.12	3.25
**IL**	4.05	3.96	4.15	3.60	3.57	3.63
**IN**	4.09	3.85	4.33	4.64	4.54	4.75
**KS**	3.51	3.19	3.84	3.34	3.25	3.43
**KY**	3.04	2.81	3.29	3.57	3.50	3.65
**LA**	4.03	3.92	4.15	5.49	5.42	5.56
**MA**	2.86	2.79	2.93	4.04	3.99	4.08
**MD**	3.43	3.28	3.59	1.98	1.92	2.05
**ME**	3.38	3.10	3.71	3.16	3.11	3.20
**MI**	2.62	2.60	2.64	2.98	2.96	3.00
**MN**	3.25	3.08	3.44	2.75	2.73	2.78
**MO**	5.17	4.74	5.63	2.36	2.28	2.46
**MS**	4.82	4.50	5.15	3.43	3.35	3.51
**MT**	3.20	2.74	3.74	3.40	3.33	3.47
**NC**	3.76	3.59	3.95	4.29	4.23	4.34
**ND**	3.79	3.08	4.50	3.83	3.74	3.92
**NE**	2.36	2.15	2.59	2.68	2.60	2.75
**NH**	2.96	2.73	3.21	2.99	2.93	3.04
**NJ**	4.74	4.66	4.83	5.75	5.66	5.83
**NM**	2.72	2.43	3.00	3.42	3.38	3.46
**NV**	3.57	3.37	3.77	3.91	3.86	3.97
**NY**	4.74	4.71	4.78	4.26	4.25	4.28
**OH**	4.12	3.92	4.32	2.88	2.79	2.97

medRxiv preprint doi: https://doi.org/10.1101/2020.04.20.20073338.this version posted April 24, 2020. The copyright holder for this preprint
(which was not certified by peer review) is the author/funder, who has granted medRxiv a license to display the preprint in perpetuity.
It is made available under a CC-BY 4.0 International license .

**OK**	3.46	3.14	3.78	3.03	2.95	3.10
**OR**	2.63	2.50	2.77	3.01	2.97	3.04
**PA**	3.75	3.62	3.87	4.25	4.20	4.30
**RI**	2.91	2.70	3.11	2.73	2.67	2.78
**SC**	3.78	3.59	4.00	3.60	3.53	3.66
**SD**	1.92	1.60	2.24	2.50	2.44	2.56
**TN**	4.05	3.89	4.20	5.88	5.80	5.97
**TX**	3.12	2.99	3.24	3.56	3.51	3.61
**UT**	3.81	3.61	4.03	4.66	4.58	4.73
**VA**	3.07	2.91	3.22	3.45	3.41	3.50
**VT**	3.95	3.55	4.37	2.75	2.69	2.80
**WA**	2.15	2.13	2.18	2.59	2.58	2.60
**WI**	3.81	3.66	3.98	4.11	4.07	4.15
**WV**	4.47	2.79	6.22	3.89	3.74	4.05
**WY**	2.89	2.43	3.43	3.12	2.97	3.29
**US**	**3.448**	**3.435**	**3.461**	**3.530**	**3.525**	**3.535**

medRxiv preprint doi: https://doi.org/10.1101/2020.04.20.20073338.this version posted April 24, 2020. The copyright holder for this preprint (which was not certified by peer review) is the author/funder, who has granted medRxiv a license to display the preprint in perpetuity.
It is made available under a CC-BY 4.0 International license .

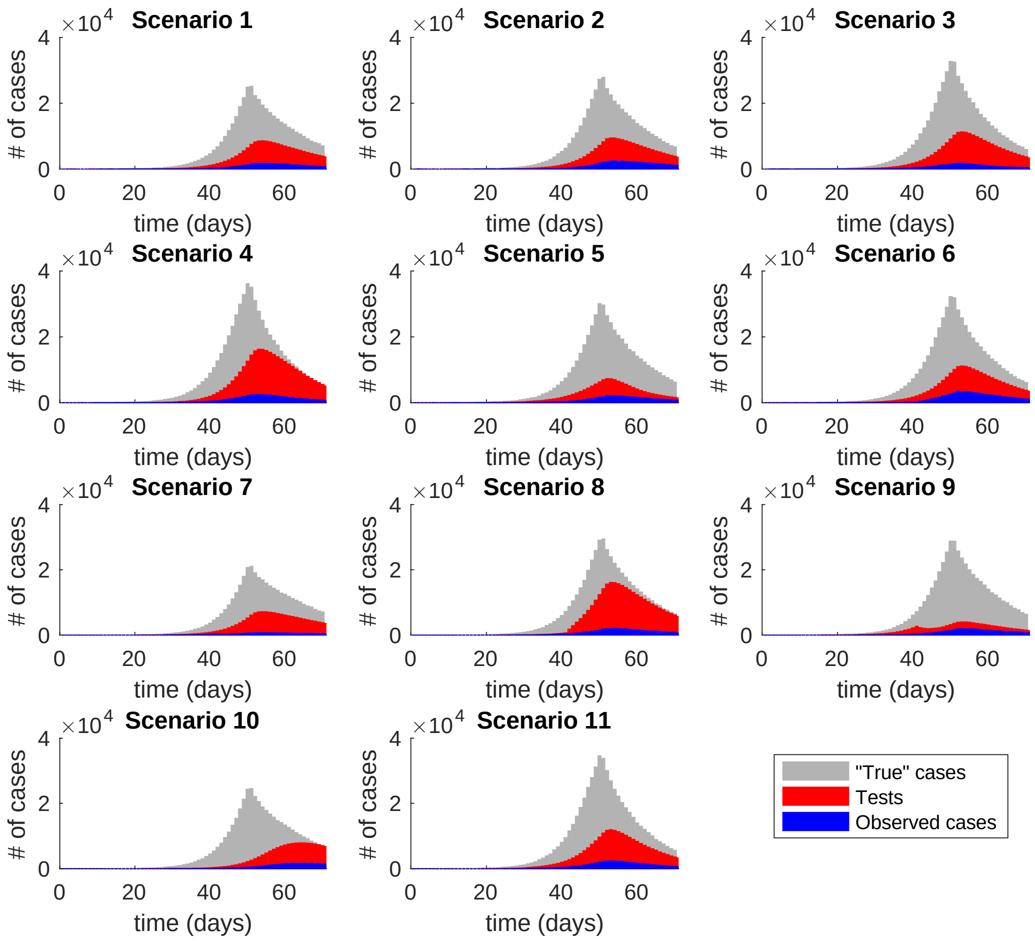

Fig. S1. Simulated epidemics for different modelled scenarios exploring changes in testing practices. Number of incident infections ("true" cases, grey), individuals tested (red), and confirmed cases (positive tests, blue) predicted by a stochastic SEIR model for a population of 1 million. Modelled scenarios are described in Table 3.

medRxiv preprint doi: https://doi.org/10.1101/2020.04.20.20073338.this version posted April 24, 2020. The copyright holder for this preprint (which was not certified by peer review) is the author/funder, who has granted medRxiv a license to display the preprint in perpetuity.
It is made available under a CC-BY 4.0 International license .

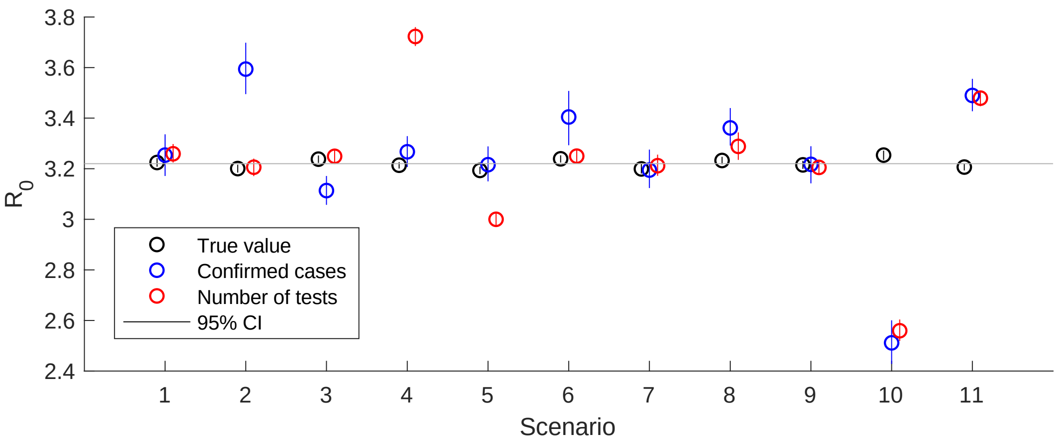

Fig. S2. Estimates of the basic reproductive number, R_0, based on simulated data. Estimated values of R_0 based on the growth rate in the number of "true" cases (black), number of confirmed cases (blue), and number of tests performed (red) are plotted for the modelled scenarios described in Table 3. The mean value is represented by the open circle, vertical lines represent 95% confidence intervals (CIs) based on uncertainty in the growth rate. The grey horizontal line represents the mean value of the "true" R_0 across all scenarios.

medRxiv preprint doi: https://doi.org/10.1101/2020.04.20.20073338.this version posted April 24, 2020. The copyright holder for this preprint
(which was not certified by peer review) is the author/funder, who has granted medRxiv a license to display the preprint in perpetuity.
It is made available under a CC-BY 4.0 International license .

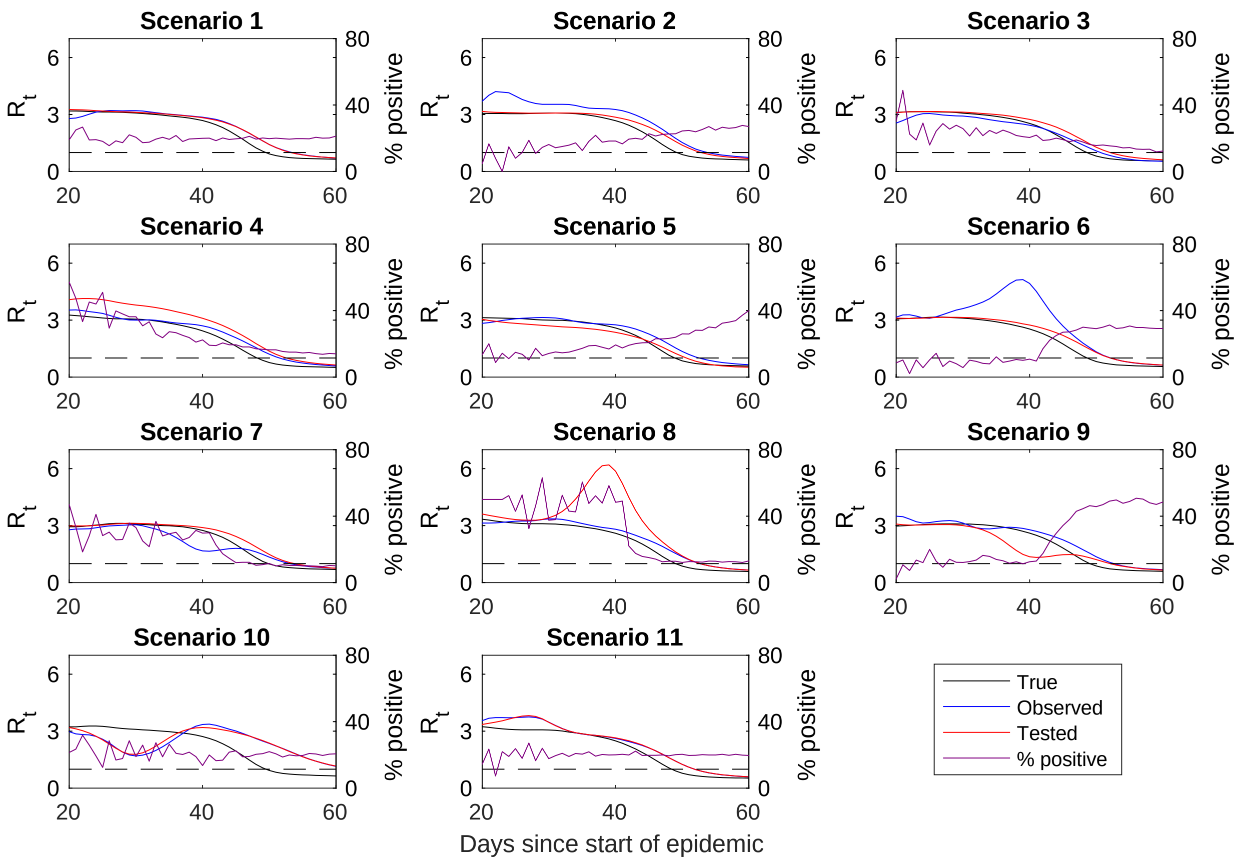

Fig. S3. Estimates of the time-varying reproductive number, R_t, based on simulated data. Values of R_t estimated by fitting to the number of "true" cases (black), confirmed cases (blue), and number of tests performed (red) are plotted along with the percent of positive tests (purple) for the modelled scenarios described in Table 3. The horizontal dashed line represents R_t=1.

medRxiv preprint doi: https://doi.org/10.1101/2020.04.20.20073338.this version posted April 24, 2020. The copyright holder for this preprint (which was not certified by peer review) is the author/funder, who has granted medRxiv a license to display the preprint in perpetuity.
It is made available under a CC-BY 4.0 International license .

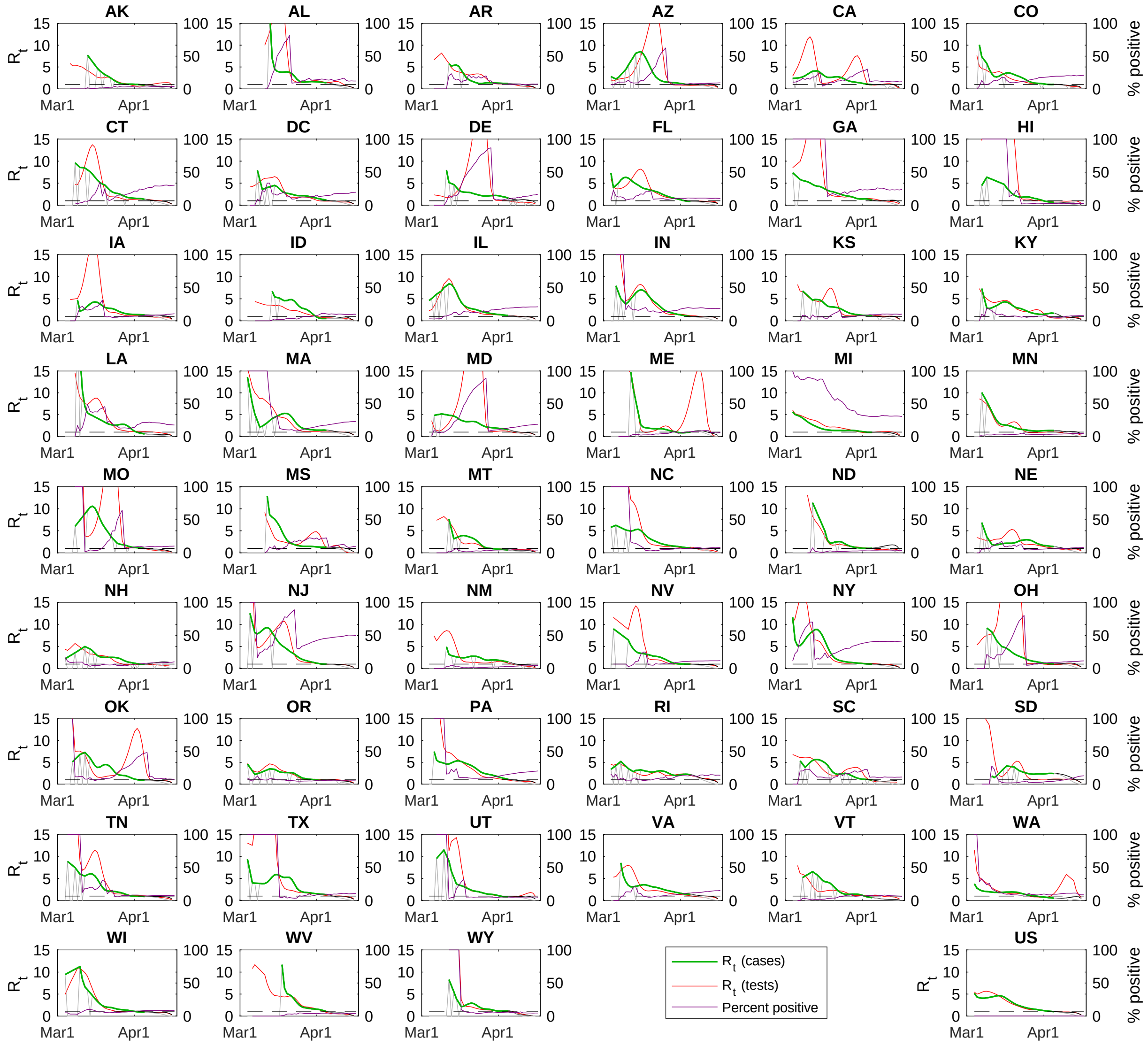

Fig. S4. Estimated time-varying reproductive numbers for 50 US states and the District of Columbia. Estimates of the time-varying reproductive number, R_t, based on the number of confirmed cases (green) and the total number of tests performed (red) are plotted for each state for March 4 to April 17, 2020, along with the percent of tests positive (purple). April 5 is the last day that the value of R_t can be reliably estimated; uncorrected (grey) and corrected (black) values of R_t are plotted for April 2 to April 16. Value of R_t based on the number of tests are corrected for right-censoring.
